# Association of ATP6V1B2 rs1106634 with lifetime risk of depression and hippocampal neurocognitive deficits: possible novel mechanisms in the etiopathology of depression

**DOI:** 10.1038/tp.2016.221

**Published:** 2016-11-08

**Authors:** X Gonda, N Eszlari, I M Anderson, J F W Deakin, G Bagdy, G Juhasz

**Affiliations:** 1 Department of Psychiatry and Psychotherapy, Kutvolgyi Clinical Center, Semmelweis University, Budapest, Hungary; 2 MTA-SE Neuropsychopharmacology and Neurochemistry Research Group, Hungarian Academy of Sciences, Semmelweis University, Budapest, Hungary; 3 Department of Pharmacodynamics, Faculty of Pharmacy, Semmelweis University, Budapest, Hungary; 4 Neuroscience and Psychiatry Unit, Institute of Brain Behaviour and Mental Health, Faculty of Medical and Human Sciences, The University of Manchester, Manchester, UK; 5 Manchester Academic Health Sciences Centre, Manchester, UK; 6 Manchester Mental Health and Social Care Partnership, Chorlton House, Manchester, UK; 7 MTA-SE-NAP B Genetic Brain Imaging Migraine Research Group, Hungarian Academy of Sciences, Semmelweis University, Budapest, Hungary

## Abstract

Current understanding and treatment of depression is limited to the monoaminergic theory with little knowledge of the involvement of other cellular processes. Genome-wide association studies, however, implicate several novel single-nucleotide polymorphisms with weak but replicable effects and unclarified mechanisms. We investigated the effect of rs1106634 of the ATPV1B2 gene encoding the vacuolar H+ATPase on lifetime and current depression and the possible mediating role of neuroticism by logistic and linear regression in a white European general sample of 2226 subjects. Association of rs1106634 with performance on frontal (Stockings of Cambridge (SOC)) and hippocampal-dependent (paired associates learning (PAL)) cognitive tasks was investigated in multivariate general linear models in a smaller subsample. The ATP6V1B2 rs1106634 A allele had a significant effect on lifetime but not on current depression. The effect of the A allele on lifetime depression was not mediated by neuroticism. The A allele influenced performance on the PAL but not on the SOC test. We conclude that the effects of variation in the vacuolar ATPase may point to a new molecular mechanism that influences the long-term development of depression. This mechanism may involve dysfunction specifically in hippocampal circuitry and cognitive impairment that characterizes recurrent and chronic depression.

## Introduction

Major depression has a great burden both in terms of suffering and costs for the society.^1, 2, 3, 4, 5^ However, its pathology is still unclear. There is a strong genetic background with risk of first-degree relatives showing a 2.8-fold increase^6, 7, 8^ and twin studies suggesting a 37–43% heritability.^7^

Due to its exceptionally large clinical heterogeneity, depression is difficult to study from a genetic point of view.^6^ Candidate gene studies select polymorphisms based on linkage studies or hypothetical choices concerning already known etiological mechanisms in the neurobiology of depression. Therefore the scope of investigated polymorphisms is rather narrow in terms of the systems in question.^9^ Consistently replicated findings from these studies are few and the identified polymorphisms confer a small amount of risk. In contrast to association studies limited to previous knowledge and researcher preferences, GWAS-s (genome-wide association studies) are more comprehensive and less biased. However, as a major drawback genome-wide significance needs to be set to very low due to the large amount of hypothesis testing. Furthermore, polymorphisms implicated in GWAS studies with at least a suggestive significance often show no connection to mechanisms already implicated in the background of depression.^6^

**Figure 1:**
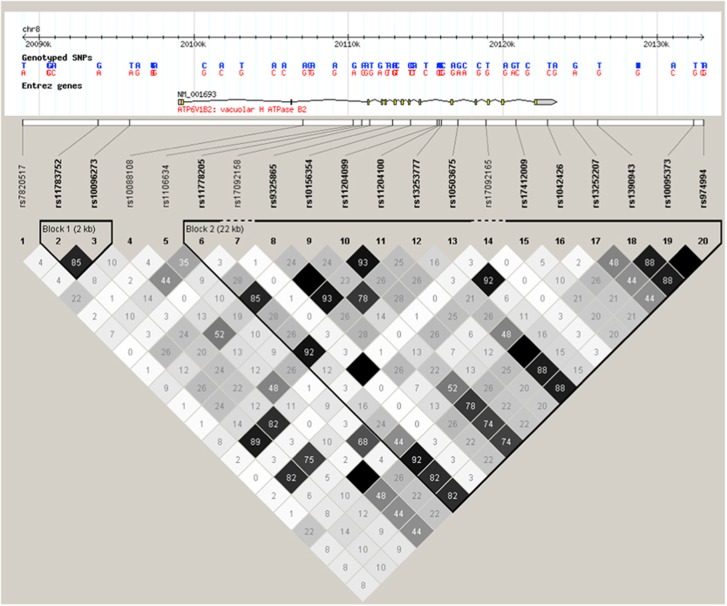
Linkage disequilibrium (LD) values (R2) and haplotype blocks of single-nucleotide polymorphisms (SNPs) in and around the *ATP6V1B2* gene. The information is based on the CEU population of version 2 and release 24 of the HapMap project (http://hapmap.ncbi.nlm.nih.gov/).

Although one novel study using a very large cohort but less intensive phenotyping based on self-reported depression identified 15 genetic loci,^10^ generally no associations with a genome-wide significance are identified in individual studies in psychiatric patients. A recent meta-analysis of three GWAS-s in major depressive disorder observed the strongest associations for three intronic single-nucleotide polymorphisms (SNPs), including one (rs1106634) in the *ATP6V1B2* gene (Figure 1) encoding the B subunit of the vacuolar H+ pump-ATPase with a suggestive significance (*P*=6.78 × 10^7^) for the A allele.^11^ In another large meta-analysis in schizophrenia and bipolar disorder, rs1106634 also exhibited a suggestive *P*-value (3.97 × 10^−6^) although for the opposite, T allele.^12^ The cytosolic domain where this subunit is located is part of a transmembrane complex playing a role in the generation of H+ gradients across synaptic vesicle membranes and is a key player in receptor-mediated endocytosis.^11^

With our current understanding and treatment approaches in depression mainly confined to monoaminergic theory and far less than adequate due to limited efficacy and significant side effects, it would be valuable to investigate SNPs with a weak but replicable effect in GWAS-s to identify new molecules of vulnerability, mechanisms of etiopathology and targets of treatment. In our study, we investigated the association of rs1106634 and depression-related phenotypes including lifetime and current depression as well as hippocampal and prefrontal-related neurocognitive deficits in a large, general, nonclinical sample in Manchester and Budapest.

## Materials and methods

This present research is part of the NewMood study, funded by the European Union (New Molecules in Mood Disorders, Sixth Framework Program of the EU, LSHM-CT-2004-503474), carried out in accordance with the Declaration of Helsinki, and approved by the local Ethics Committees (Scientific and Research Ethics Committee of the Medical Research Council, Budapest, Hungary; and North Manchester Local Research Ethics Committee, Manchester, UK).

### Participants

All participants provided written informed consent. Our study involved two levels, 1 and 2. Level 1 sample was recruited through advertisements and general practices in Budapest, and through advertisements, general practices and a website in Greater Manchester. *N*=2226 subjects (*n*=904 in Budapest and *n*=1322 in Manchester) were successfully genotyped for *ATP6V1B2* rs1106634, and provided information about gender, age, lifetime and current depression by filling out the NewMood questionnaire pack.^13^ All Level 1 subjects were of white European ethnic origin, and had no relatives participating.

Level 2 sample was collected in Manchester. *N*=206 subjects (*n*=114 having participated in Level 1; and *n*=92 new subjects recruited through advertisements) were successfully genotyped for *ATP6V1B2* rs1106634 and interviewed for Montgomery-Åsberg Depression Rating Scale and lifetime depression, provided information about gender, age and neuroticism, and carried out both cognitive tasks.

### Phenotypic assessment

Level 1 questionnaire pack included the Brief Symptom Inventory (BSI),^14^ the Big Five Inventory (BFI-44)^15^ and a background questionnaire containing a question about lifetime depression, validated with face-to-face diagnostic interviews in a subsample.^13^ We calculated a continuous weighted score (sum of item scores divided by the number of items completed) for the BSI depression subscale (depression items plus additional items) (BSI-Dep), and for the BFI-44 neuroticism scale and used these variables for the analyses.

In Level 2, we measured lifetime depression with a clinical diagnosis of major depressive disorder: trained researchers administered the Structured Clinical Interview for DSM-IV (SCID-I/NP).^16^ Those with bipolar disorder or any other psychiatric condition except for major depression were excluded from the analyses. Montgomery-Åsberg Depression Rating Scale (MADRS) was used to measure depressive symptoms.^17^ Neuroticism was measured with the neuroticism subscale of the NEO-PI-R.^18^

In Level 2, to assess neurocognitive endophenotypes of depression, we chose two computerized tasks from the Cambridge Neuropsychological Test Automated Battery software (CANTAB; http://www.cambridgecognition.com/). Paired associates learning (PAL) task assesses episodic memory, thus medial temporal lobe functioning, by requiring the subject to learn paired associations between a visual pattern and a spatial location (http://www.cambridgecognition.com/tests/paired-associates-learning-pal), and indicated a hippocampal dysfunction in depression^19^ and other neuropsychiatric disorders.^20, 21^ Three scores were calculated and used in our analyses: memory score on the first trial (PAL memory score), total number of errors (PAL errors) and total number of trials (PAL required trials). The other task, Stockings of Cambridge (SOC) measures frontal lobe function by a test of spatial planning and spatial working memory (http://www.cambridgecognition.com/tests/stockings-of-cambridge-soc), and is sensitive to executive deficits in depression.^19, 22^ We calculated and analyzed two scores of SOC: mean of all initial thinking times throughout the SOC task (SOC ITT), and rate of problems solved in minimum moves (SOC correct trial rate).

### Genotyping

Participants provided buccal mucosa cells using a genetic saliva sampling kit. We extracted genomic DNA according to Freeman *et al.*^23^ *ATP6V1B2* rs1106634 was genotyped with Sequenom MassARRAY technology (Sequenom, San Diego, CA, USA www.sequenom.com). All laboratory work was performed under ISO9001:2000 quality management requirements, and blinded for phenotype.

### Statistical analyses

We used PLINK v1.07 (http://pngu.mgh.harvard.edu/purcell/plink/) to calculate Hardy–Weinberg equilibrium and to build regression models in Level 1. Logistic regression equations were run on lifetime depression, and linear regressions on BSI depression score, in additive, dominant and recessive models, with *ATP6V1B2* rs1106634, population (Budapest or Manchester), gender and age as predictors. Bonferroni-corrected significance threshold was *P*⩽0.0083 for these six analyses. As *post hoc* tests in Level 1, with a nominal *P*⩽0.05 significance threshold, we intended to replicate the significant findings in the Budapest and Manchester subsamples, separately. Also as *post hoc* tests we tested the putative mediating effect of neuroticism in the combined Level 1 Budapest+Manchester sample for the significant findings, including it as an additional predictor in the regression equations.

IBM SPSS Statistics 23 was used for descriptive statistics, and to test *post hoc* effect of *ATP6V1B2* rs1106634 on PAL and SOC performance in Level 2. We built a multivariate general linear model for the three PAL outcomes, and another one for the two SOC outcomes, with *ATP6V1B2* rs1106634 as a fixed factor and gender, age, lifetime depression, MADRS score and NEO-PI-R neuroticism score as covariates in both models. A nominal *P*⩽0.05 significance threshold was applied. Similar multivariate models, but without rs1106634, were used to gain residuals for the task performance variables in order to visualize the genetic effect on them in the figures.

Quanto (http://biostats.usc.edu/Quanto.html) was used to calculate the power of the main six tests, giving the Bonferroni-corrected *P*⩽0.0083 as type I error rate. Power to detect main genetic effects on lifetime depression is moderate or low: assuming an odds ratio (OR) of 1.2 and a population prevalence of 15% for lifetime depression, the power in our sample is 50.31% in a log-additive, 29.98% in a dominant and 5.79% in a recessive model. The power is high to detect genetic main effects on BSI depression score (of which mean is 0.87 and s.d. is 0.931 in our sample and *n*=2222, assuming an *R*^2^=1%): the power is 98.15% in all three models.

Individually written R-scripts^24^ were applied in the PLINK analysis of the Level 1 subsamples to perform the separate analyses in the Budapest and Manchester subsamples.

## Results

### Description of the sample

Rs1106634 A is the minor allele in our sample. Our sample was in Hardy–Weinberg equilibrium (*P*>0.05). Description of the Level 1 and 2 samples is shown in Table 1. The Level 1 Budapest and Manchester subsamples differ significantly in all variables except for gender, so including population as a predictor in the regression equations is required.

### Effect of the A allele on lifetime and current depression

In the combined Level 1 sample, *ATP6V1B2* rs1106634 A allele exerts a Bonferroni-corrected significant effect as a risk for lifetime depression in additive and dominant, but not in recessive models (Table 2). Nevertheless, its effect is not significant on BSI depression score in any of the models (Table 2). *Post hoc* analyses indicated that the risk of the A allele on lifetime depression is replicable in the two subsamples (Table 3).

### The mediatory role of neuroticism

We investigated whether the effect of *ATP6V1B2* rs1106634 A allele on lifetime depression is due to the association of the A allele with higher neuroticism scores. In the combined Level 1 sample, with population, gender and age as covariates, A allele is in a significant positive association with neuroticism score (its *β*=0.102; *t*=2.724; *P*=0.006 in an additive, and *β*=0.114; *t*=2.721; *P*=0.007 in a dominant model). Lifetime depression also associates positively to neuroticism (*t*=−27.996; *P*<0.001): mean neuroticism score is 3.70±0.026 in those who did, and 2.75±0.022 in those who did not report lifetime depression. So, we could test the possible mediating role of neuroticism in the effect of the A allele on lifetime depression risk. As *post hoc* tests with a nominal *P*⩽0.05 significance threshold, including neuroticism as an additional covariate in the regression equations, besides population, gender and age, the significant positive effect of A allele on lifetime depression risk decreased but did not disappear: OR=1.317; *t*=2.562; *P*=0.010 in the additive, and OR=1.367; *t*=2.622; *P*=0.009 in the dominant model (for comparisons, see Table 2), indicating that the effect of the A allele on depression is not mediated solely by the association of this allele with neuroticism.

### Association of the A allele with brain region-specific cognitive performance

**Figure 2:**
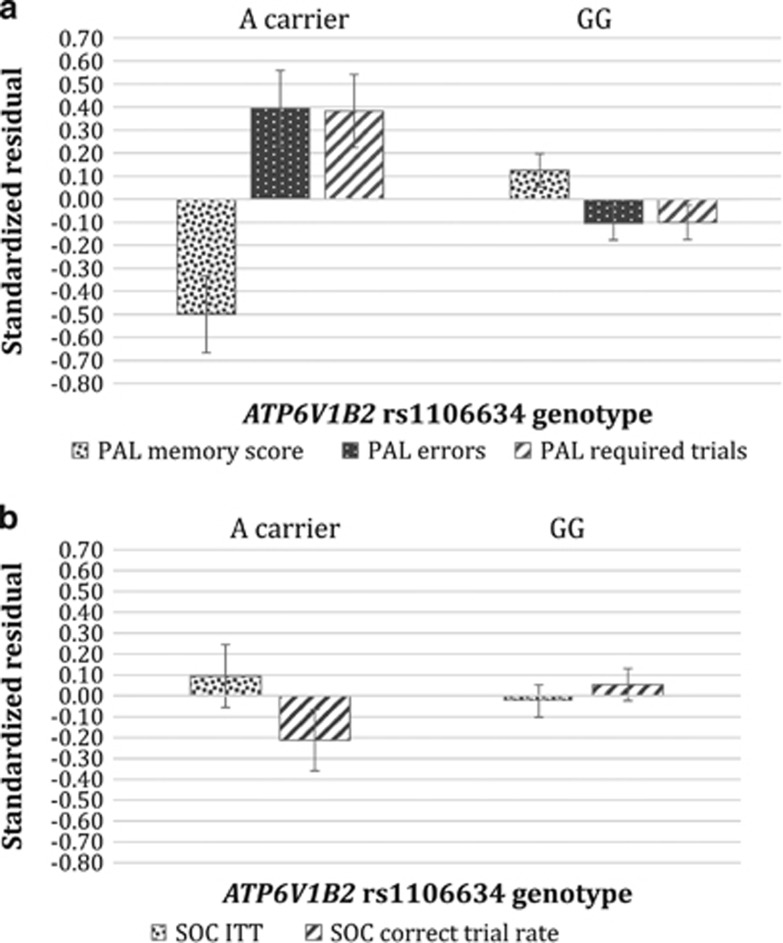
Multivariate general linear models indicate a significant effect of ATP6V1B2 rs1106634 genotype on PAL task performance but not on SOC task performance. Multivariate general linear models for the three PAL variables (**a**) and the two SOC variables (**b**) with gender, age and lifetime depression, current (MADRS) depression and neuroticism as covariates. Means of standardized residuals for the PAL (**a**) and SOC (**b**) variables are displayed in function of ATP6V1B2 rs1106634 genotype. A allele carriers show a significantly lower memory score on the first trial (*P*<0.001), but a significantly higher total number of errors (*P*=0.003) and required trials (*P*=0.004) than those with GG genotype in the PAL task (**a**). However, the two genotype groups (A allele carriers and GG carriers) do not differ in the two aspects of SOC performance (SOC ITT *P*=0.435, SOC correct trial rate *P*=0.155) (**b**). PAL, paired associates learning task; PAL errors, total number of errors on PAL; PAL memory score, memory score on the first trial of PAL; PAL required trials, total number of trials on PAL; SOC, Stockings of Cambridge task; SOC correct trial rate, rate of problems solved in minimum moves; SOC ITT, mean of all initial thinking times throughout the SOC task. Means (and the s.e.m.) of standardized residuals for the three PAL (**a**) and the two SOC (**b**) scores, partialling out variance accounted for by gender, age, lifetime depression, MADRS and neuroticism, are represented according to genotype.

Next, we tested *post hoc*, in Level 2, if *ATP6V1B2* rs1106634 A allele explains the variance of performance on the two neurocognitive tasks independently of the variance explained by lifetime depression, current depression and neuroticism. A multivariate general linear model was built with A allele as a fixed factor (in a dominant model, because of low number in the AA group, see Table 1), gender, age, lifetime depression, MADRS and neuroticism as covariates, and PAL memory score, PAL errors and PAL required trials as the dependent variables. Wilks' Lambda of the A allele showed *F*=5.661, *P*=0.001. The A allele exerted a nominally significant effect on each PAL variable in the model (PAL memory: *F*=15.159; *P*<0.001; PAL errors: *F*=9.350; *P*=0.003; PAL required trials: *F*=8.546; *P*=0.004). Another multivariate general linear model was built with the A allele as a fixed factor (in a dominant model), gender, age, lifetime depression, current depression (MADRS) and neuroticism as covariates, and SOC ITT and SOC correct trial rate as dependent variables. For Wilks' Lambda of the A allele, *F*=1.665; *P*=0.192. In this model, the A allele did not exert a significant effect on any of the SOC variables (SOC ITT: *F*=0.612; *P*=0.435; SOC correct trial rate: *F*=2.039; *P*=0.155; Figure 2).

## Discussion

In our study in a large general sample *ATP6V1B2* rs1106634 A allele was a significant risk factor for lifetime major depression but was not associated with current depressive symptom manifestation. This risk effect of the A allele on depression was only partially mediated by neuroticism, a well-known risk factor for depression, which was associated with both depression and the A allele in our study indicating overlapping genetic risk but different mechanisms. Furthermore, our results show that rs1106634 may exert a brain region-specific effect independently of lifetime history or current level of depression impacting temporal-hippocampal but not frontal functions as reflected by significantly decreased performance in A allele carriers on the PAL but not on the SOC neurocognitive test. Our results thus show that *ATP6V1B2* rs1106634 may play an important role in the long-term development and risk for depression, but not in its cross-sectional symptomatology, possibly via its effect on hippocampal function conceived to play a central role in depression.

### Association of rs1106634 A allele with risk for lifetime depression but not current depressive symptoms

In line with previous GWAS-s implicating the involvement of the *ATP6V1B2* rs1106634 in affective disorders,^11, 12, 25^ we found a significant association between the A allele and lifetime depression in our large, average population of 2226 subjects and replicated our findings in two independent subsamples from Budapest and Manchester showing the involvement of this variant in the lifetime risk for depression. Interestingly, we found no association between this polymorphism and current depressive symptoms, indicating that this genetic variant is not associated directly with currently detectable symptoms but may be related to cellular mechanisms associated with the development of depression on brain functional or structural levels. On the basis of our findings, rs1106634 is involved in the development of a trait-like risk for depression rather than its state-like manifestation. Furthermore, it is possible that consequences of the presence of A allele are manifested in neurocognitive rather than mood symptoms of depression which are hypothesized to play a central role in depression but are not captured by conventional rating scales, and can be only detected by assessing neurocognitive performance, as indicated in our study by the significantly lower performance on the PAL test reflecting impaired hippocampal function in A allele carriers.

### Association of rs1106634 with lifetime risk of depression is not mediated by neuroticism

The rs1106634 A allele was significantly associated with neuroticism, a well-established prospective risk factor for depression.^26^ Neuroticism is a personality trait of negative emotionality associated with less adaptive coping with inner and outer stressors and increased mood lability leading to a heightened risk of experiencing anxiety and depressed mood.^27^ Therefore, its presence is likely to play a developmental role in depression, which further supports our notion that the A allele influences long-term adjustment and trait-like risk factors rather than state-like symptomatic presentation of depression. Although neuroticism is one of the most widely studied endophenotypes of depression with about 55% of shared genetic risk,^28^ we found that neuroticism only partially mediated the effects of rs1106634 on lifetime depression. We conclude that rs1106634 may influence neuroticism and depression in ways that are partially independent.

### Association of rs1106634 with hippocampal but not prefrontal neurocognitive dysfunction

In our study, rs1106634 A allele was associated with hippocampal cognitive deficits indicated by decreased performance on all measures of the PAL task independently of lifetime or current depression and neuroticism, while prefrontal functions indicated by the SOC test remained intact. Thus our results raise the possibility that the effect of this polymorphism is area-specific affecting temporal rather than prefrontal lobe function. Given the role of the vacuolar ATPase in cellular function and the fact that the hippocampus is an area with high energy and oxygen need and increased vulnerability compared with other regions,^29, 30, 31^ it is possible that this area is more sensitive to possible functional consequences of this polymorphic variant.

PAL performance involves learning random visuospatial associations and is sensitive to hippocampal damage.^21^ Michopoulos *et al.* reported that impaired PAL was associated with measures of the severity and chronicity of depression including illness duration and number of prior episodes. In contrast in the same study no such measures of depression chronicity were found to be associated with SOC scores.^19^ This is in line with our assumption that rs1106634 effects are reflected in lifetime rather than acute depression measures and are linked to long-term risk and development of depression.

Our results indicating a specific association between hippocampal dysfunction and rs1106634 are especially interesting as hippocampal dysfunction is thought to play a central role as part of a larger dysregulated circuitry implicated in the pathophysiology of depression.^32, 33^ Loss of hippocampal volume is associated with several measures of increased long-term depression severity and worse clinical outcome^33^ and structural effects of genetic influences.^34^ The hippocampus is considered a brain region with increased sensitivity to stress^35^ resulting in structural changes as well as enhanced vulnerability to metabolic insults^33^ and depression is also a highly stress-associated illness. Therefore, the possible consequences of genetically determined differences in vacuolar H+ATPase may be especially manifested in the most vulnerable regions such as the hippocampus in high stress-associated illnesses such as depression.

### Possible mechanisms behind the role of rs1106634 in depression and hippocampal dysfunction

Rs1106634 is located in the intron of the *ATP6V1B2* gene, encoding the B subunit of the ATP-catalytic site containing the V1 cytosolic domain of the vacuolar proton pump-ATPase,^11^ which mediates acidification of various eukaryotic intracellular organelles such as lysosomes and endosomes, necessary for processes including protein sorting, receptor-mediated endocytosis and synaptic vesicle proton-gradient generation. The protonmotive force generated by vacuolar ATPases is used for various secondary reactions including synaptic vesicular reuptake, accumulation and storage.^36, 37^ Transport including retrieval and reemployment of synaptic vesicles is an essential component in the management and maintenance of neurotransmission. The vacuolar H+ATPase plays a key role in the energization of regenerated synaptic vesicles after endocytosis, and thus in providing power for the replenishment of neurotransmitters via specific vesicular transporters.^37, 38^ Therefore generation of the electrochemical H+ gradient is essential and even its minor or partial disturbance may cause altered quantal neurotransmitter release.^38, 39^ Vacuolar ATPase inhibition was found to cause downregulation in important signaling pathways including Wnt and Notch, although it has not yet been clarified to what extent this effect is a consequence of its function in endosome acidification or whether it is due to its effect on endocytosis and trafficking.^40^ Vacuolar ATPase was also found to maintain neural stem cells in the developing cortex of mice.^40^ Although the role of vacuolar ATPase in central nervous system function is becoming clearer, no specific effect of this polymorphism on neuronal function has been specifically investigated.

One possible mechanism by which variations in the vacuolar ATPase may exert their effect on depression risk is its role in oxidative stress, also implicated in the etiopathology of depression. Emotional stressors appear to induce inflammatory responses which are in turn associated with increased proinflammatory cytokine production, stimulating reactive oxygen and nitrogen species production and leading to oxidative and nitrosative stress, free radical production and brain damage by increasing neurodegeneration and impairing neurogenesis.^41^ Chronic inflammation as well as oxidative and nitrosative stress has long been hypothesized to play a role in recurrent major depression and especially in neurocognitive deficits.^31, 41^ Cellular redox status may play a role in the regulation of the vacuolar H+ATPase hypothesized to be a redox sensor regulating neurotransmitter storage in response to growth factor signaling or oxidative stress, leading to decreased neurotransmitter amounts in vesicles and therefore decreased or diminished neurotransmitter release.^42^ In a more recent GWAS, a suggestive association between serum myeloperoxidase levels and a region on chromosome 8p21.3 containing *ATP6V1B2* genes was reported.^43^ Myeloperoxidase is a surrogate marker of prooxidative stress in major depression, and its serum levels were previously associated with depression.^44^ Myeloperoxidase expression was also reported to be associated with neurodegeneration, higher risk of Alzheimer's disease,^45^ neuronal cell death and neurogenesis inhibition.^41^

The brain shows enhanced vulnerability to oxidative damage as it uses large amounts of oxygen. Lipids, including unsaturated fatty acids, which are especially reactible with free radicals are abundant in central nervous system cells. Furthermore, several brain regions are high in metal ions that promote reactive oxygen species formation, and lower antioxidant concentrations are observable in the brain compared with other body organs.^41^ The most sensitive to oxidative damage in the central nervous system are, among others, CA1 and CA4 hippocampal regions as well as the dorsal lateral striatum and III and V cortex layers.^31^ Especially CA1 neurons are highly vulnerable, playing a role in long-term memory and spatially related tasks,^30^ which corresponds to the deficit seen in our study.

### *In silico* functional analysis of rs1106634 and comparison with results of the Psychiatric Genetic Consortium GWAS

*In silico* functional prediction using the SNP Function Prediction Tool (http://snpinfo.niehs.nih.gov/snpinfo/snpfunc.htm) showed no functional effects of our investigated SNP rs1106634, however, a nearby SNP with an LD of 0.62, namely the rs1042426, is a microRNA binding site, which suggests that this genetic region may have functional consequences related to gene expression regulation. Furthermore, the latest mega-analysis of the Psychiatric Genetic Consortium demonstrated that SNPs in high LD (based on the CEU population data) with rs1106634, namely rs6586899 (*r*^2^=0.96) and rs4335136 (*r*^2^=0.86), showed nominally significant associations with major depressive disorder (rs6586899 *P*=0.004, rs4335136 *P*=0.017), while the functional rs1042426 has a trend effect (*P*=0.089) supporting the weak but replicable role of *ATP6V1B2* in lifetime depression.^46^

### Limitations

Several limitations of our study must be noted. We measured depressive symptoms and neurocognitive performance cross-sectionally. Both current and lifetime depression was based on self-report thus could be influenced by reporting and recalling bias. Recent life events possibly influencing current depression as well as general intellectual performance possibly impacting neurocognitive performance were not included in our models. Furthermore, our study sample is a general, non-epidemiological and non-representative population sample based on volunteers, therefore may be subject to sampling bias especially with respect to depression.

### Conclusion and implication for further studies

The main finding of our study is supporting the role of the *ATP6V1B2* rs1106634 A allele, implicated in depression GWAS-s with a suggestive significance, as a risk allele for lifetime but not current depression suggesting a role in the long-term development of affective illness. This finding is further underlined by the association of this SNP with neuroticism, a well-established risk factor of depression, while the fact that neuroticism only partially mediated the association between rs1106634 and depression indicates a differential involvement of rs1106634 in these two phenomena. Our results also show that the effect of rs1106634 on lifetime risk of depression may be due to the association of the A allele with hippocampal dysfunction, thought to play a central role in the development of depression. We must also mention that while rs1106634 was implicated in several GWAS-s in psychiatric patients, it was not among the 15 genetic loci identified in major depression in a most recent study using an extremely large cohort with less intensive phenotyping approach using self-reported lifetime depression,^10^ the same phenotype as used in our study.

On the clinical level, our findings concerning the role of the vacuolar ATPase in depression and depression-associated cognitive deficits suggest that pharmacological interventions targeting this molecule may be promising new molecular agents in the treatment of depression in general and also in cases with more marked neurocognitive symptoms, also possibly able to tackle the progressive and degenerative nature of such symptoms. Our results thus emphasize the importance of looking beyond the monoaminergic systems in investigating mechanisms underlying depression in order to establish a more accurate model of this illness and pinpoint possible targets for pharmacological intervention, and also stress the importance of investigating polymorphisms implicated in depression GWAS-s, even if only with a weak effect, with respect to their possible role and mechanism.

## Figures and Tables

**Table 1:** Description of the samples and subsamples

		*Level 1*					*Level 2*
		*Budapest*	*Manchester*	*Budapest + Manchester*	*Difference between Budapest and Manchester*	*P-values*	*Manchester*
Gender	Female (%)	631 (69.8%)	922 (69.7%)	1553 (69.8%)	*χ*^2^=0.001	0.977	142 (68.9%)
	Male (%)	273 (30.2%)	400 (30.3%)	673 (30.2%)			64 (31.1%)
*ATP6V1B2* rs1106634	AA (%)	12 (1.3%)	28 (2.1%)	40 (1.8%)	*χ*^2^=10.445	0.005	7 (3.4%)
	GA (%)	184 (20.4%)	337 (25.5%)	521 (23.4%)			35 (17.0%)
	GG (%)	708 (78.3%)	957 (72.4%)	1665 (74.8%)			164 (79.6%)
Lifetime depression	Reported (%)	197 (21.8%)	740 (56.0%)	937 (42.1%)	*χ*^2^=257.378	<0.001	108 (52.4%)
	Not reported (%)	707 (78.2%)	582 (44.0%)	1289 (57.9%)			98 (47.6%)
Age (mean±s.e.m.)		31.24 (0.353)	34.03 (0.282)	32.90 (0.222)	*t*=−6.216	<0.001	32.41 (0.730)
BSI depression score (mean±s.e.m.)		0.57 (0.023)	1.07 (0.028)	0.87 (0.020)	*t*=−13.744	<0.001	
MADRS depression score (mean±s.e.m.)							4.58 (0.493)
Neuroticism score (mean±s.e.m.)		2.84 (0.028)	3.36 (0.025)	3.15 (0.019)	*t*=−13.922	<0.001	93.04 (1.957)
PAL memory score (mean±s.e.m.)							21.03 (0.251)
PAL errors (mean±s.e.m.)							7.57 (0.625)
PAL required trials (mean±s.e.m.)							10.76 (0.219)
SOC ITT (mean±s.e.m.) (ms)							5709.34 (306.745)
SOC correct trial rate (mean±s.e.m.)							0.74 (0.011)

Abbreviations: BSI, Brief Symptom Inventory; MADRS, Montgomery-Åsberg Depression Rating Scale; PAL, Paired associates learning task; PAL memory score, memory score on the first trial of PAL; PAL errors, total number of errors on PAL; PAL required trials, total number of trials on PAL; *χ*^2^, Pearson chi-square; s.e.m., standard error of mean; SOC, Stockings of Cambridge task; SOC correct trial rate, rate of problems solved in minimum moves; SOC ITT, mean of all initial thinking times throughout the SOC task.

**Table 2:** Main effect of *ATP6V1B2* rs1106634 on the two depression phenotypes in our Level 1 sample (Manchester and Budapest combined sample)

	*Lifetime depression*					*BSI depression score*				
*Model*	N	*OR*	*95% CI*	t	P*-values*	N	β	*s.e.*	t	P*-values*
Additive	**2226**	**1.406**	**1.165–1.696**	**3.560**	**<0.001**	2222	0.048	0.039	1.214	0.225
Dominant	**2226**	**1.465**	**1.189–1.804**	**3.591**	**<0.001**	2222	0.042	0.044	0.965	0.335
Recessive	2226	1.527	0.779–2.993	1.234	0.217	2222	0.179	0.142	1.255	0.210

Abbreviations: 95% CI, 95% confidence interval of OR; BSI, Brief Symptom Inventory; OR, odds ratio; *P*, nominal *P*-value; s.e., standard error of *β*. Population (Budapest or Manchester), gender and age were covariates in all of the six regression equations. The three models correspond to that A is the minor allele in all cases. Significant findings according to the Bonferroni-corrected threshold (*P*⩽0.0083) are marked with bold.

**Table 3:** Main effect of *ATP6V1B2* rs1106634 on lifetime depression in the two Level 1 subsamples in Manchester and Budapest

	*Budapest*					*Manchester*				
*Model*	N	*OR*	*95% CI*	t	P*-values*	N	*OR*	*95% CI*	t	P*-values*
Additive	904	1.541	1.109–2.142	2.574	0.010	1322	1.342	1.070–1.682	2.549	0.011
Dominant	904	1.628	1.129–2.348	2.609	0.009	1322	1.387	1.079–1.784	2.549	0.011

Abbreviations: 95% CI, 95% confidence interval of OR; OR, odds ratio; *P,* nominal *P*-value. Gender and age were covariates in all of the four regression equations. The models correspond to that A is the minor allele in both subsamples. All findings are nominally significant (*P*⩽0.05).

## References

[bib1] Murray CJ, Lopez AD. Global mortality, disability, and the contribution of risk factors: Global Burden of Disease Study. Lancet 1997; 349: 1436–1442. 9164317 10.1016/S0140-6736(96)07495-8

[bib2] Murray CJL, Lopez AD. The Global Burden Of Disease: A Comprehensive Assessment of Mortality and Disability From Diseases, Injuries, and Risk Factors in 1990 and Projected to 2020. Harvard School of Public Health on behalf of the World Health Organization and the World Bank; Distributed by Harvard University Press: Cambridge, MA, 1996.

[bib3] Mathers C, Fat DM, Boerma JT, World Health Organization. The Global Burden of Disease: 2004 Update. World Health Organization: Geneva, Switzerland, 2008.

[bib4] Wittchen HU. The burden of mood disorders. Science 2012; 338: 15–.. 23042853 10.1126/science.1230817

[bib5] Kessler RC, Berglund P, Demler O, Jin R, Koretz D, Merikangas KR et al. The epidemiology of major depressive disorder - Results from the National Comorbidity Survey Replication (NCS-R). JAMA 2003; 289: 3095–3105. 12813115 10.1001/jama.289.23.3095

[bib6] Shyn SI, Hamilton SP. The genetics of major depression: moving beyond the monoamine hypothesis. Psychiatr Clin North Am 2010; 33: 125–140. 20159343 10.1016/j.psc.2009.10.004 PMC2824618

[bib7] Sullivan PF, Neale MC, Kendler KS. Genetic epidemiology of major depression: review and meta-analysis. Am J Psychiatry 2000; 157: 1552–1562. 11007705 10.1176/appi.ajp.157.10.1552

[bib8] Holmans P, Zubenko GS, Crowe RR, DePaulo JR Jr, Scheftner WA, Weissman MM et al. Genomewide significant linkage to recurrent, early-onset major depressive disorder on chromosome 15q. Am J Hum Genet 2004; 74: 1154–1167. 15108123 10.1086/421333 PMC1182079

[bib9] Lopez-Leon S, Janssens AC, Gonzalez-Zuloeta Ladd AM, Del-Favero J, Claes SJ, Oostra BA et al. Meta-analyses of genetic studies on major depressive disorder. Mol Psychiatry 2008; 13: 772–785. 17938638 10.1038/sj.mp.4002088

[bib10] Hyde CL, Nagle MW, Tian C, Chen X, Paciga SA, Wendland JR et al. Identification of 15 genetic loci associated with risk of major depression in individuals of European descent. Nat Genet 2016; 48: 1031–1036. 27479909 10.1038/ng.3623 PMC5706769

[bib11] Shyn SI, Shi J, Kraft JB, Potash JB, Knowles JA, Weissman MM et al. Novel loci for major depression identified by genome-wide association study of Sequenced Treatment Alternatives to Relieve Depression and meta-analysis of three studies. Mol Psychiatry 2011; 16: 202–215. 20038947 10.1038/mp.2009.125 PMC2888856

[bib12] Wang KS, Liu XF, Aragam N. A genome-wide meta-analysis identifies novel loci associated with schizophrenia and bipolar disorder. Schizophr Res 2010; 124: 192–199. 20889312 10.1016/j.schres.2010.09.002

[bib13] Juhasz G, Dunham JS, McKie S, Thomas E, Downey D, Chase D et al. The CREB1-BDNF-NTRK2 pathway in depression: multiple gene-cognition-environment interactions. Biol Psychiatry 2011; 69: 762–771. 21215389 10.1016/j.biopsych.2010.11.019

[bib14] Derogatis LR. Brief Symptom Inventory: Administration, Scoring, and Procedures Manual. National Computer Systems Pearson, Inc.: Minneapolis, MN, 1993.

[bib15] John OP, Donahue EM, Kentle RL. The Big Five Inventory: Versions 4a and 54. Technical Report. University of California. Insititute of Personality and Social Research: Berkeley, 1991.

[bib16] First MB, Spitzer RL, Gibbon M, Williams JBW. Structured Clinical Interview for DSM-IV-TR Axis I Disorders, Research Version (SCID-I). State Psychiatric Institute: New York, 2002.

[bib17] Montgomery SA, Asberg M. A new depression scale designed to be sensitive to change. Br J Psychiatry 1979; 134: 382–389. 444788 10.1192/bjp.134.4.382

[bib18] Costa PT, McCrea RR. Revised NEO Personality Inventory (NEO PI-R) and NEO Five-Factor Inventory (NEO-FFI). Psychological Assessment Resources: Odessa, FL, 1992.

[bib19] Michopoulos I, Zervas IM, Pantelis C, Tsaltas E, Papakosta VM, Boufidou F et al. Neuropsychological and hypothalamic-pituitary-axis function in female patients with melancholic and non-melancholic depression. Eur Arch Psychiatry Clin Neurosci 2008; 258: 217–225. 18297425 10.1007/s00406-007-0781-8

[bib20] Siraly E, Szabo A, Szita B, Kovacs V, Fodor Z, Marosi C et al. Monitoring the early signs of cognitive decline in elderly by computer games: an MRI study. PLoS One 2015; 10: e0117918. 25706380 10.1371/journal.pone.0117918 PMC4338307

[bib21] Blackwell AD, Sahakian BJ, Vesey R, Semple JM, Robbins TW, Hodges JR. Detecting dementia: novel neuropsychological markers of preclinical Alzheimer's disease. Dement Geriatr Cogn Disord 2004; 17: 42–48. 14560064 10.1159/000074081

[bib22] Elliott R, Sahakian BJ, McKay AP, Herrod JJ, Robbins TW, Paykel ES. Neuropsychological impairments in unipolar depression: the influence of perceived failure on subsequent performance. Psychol Med 1996; 26: 975–989. 8878330 10.1017/s0033291700035303

[bib23] Freeman B, Smith N, Curtis C, Huckett L, Mill J, Craig IW. DNA from buccal swabs recruited by mail: evaluation of storage effects on long-term stability and suitability for multiplex polymerase chain reaction genotyping. Behav Genet 2003; 33: 67–72. 12645823 10.1023/a:1021055617738

[bib24] R Core TeamR: A Language and Environment for Statistical Computing. R Foundation for Statistical Computing: Vienna, 2013.

[bib25] Sklar P, Smoller JW, Fan J, Ferreira MAR, Perlis RH, Chambert K et al. Whole-genome association study of bipolar disorder. Mol Psychiatry 2008; 13: 558–569. 18317468 10.1038/sj.mp.4002151 PMC3777816

[bib26] Clark LA, Watson D, Mineka S. Temperament, personality, and the mood and anxiety disorders. J Abnorm Psychol 1994; 103: 103–116. 8040472

[bib27] Klein DN, Kotov R, Bufferd SJ. Personality and depression: explanatory models and review of the evidence. Annu Rev Clin Psychol 2011; 7: 269–295. 21166535 10.1146/annurev-clinpsy-032210-104540 PMC3518491

[bib28] Levinson DF. The genetics of depression: a review. Biol Psychiatry 2006; 60: 84–92. 16300747 10.1016/j.biopsych.2005.08.024

[bib29] Nishino H, Hida H, Kumazaki M, Shimano Y, Nakajima K, Shimizu H et al. The striatum is the most vulnerable region in the brain to mitochondrial energy compromise: a hypothesis to explain its specific vulnerability. J Neurotrauma 2000; 17: 251–260. 10757330 10.1089/neu.2000.17.251

[bib30] Wang XK, Michaelis EK. Selective neuronal vulnerability to oxidative stress in the brain. Front Aging Neurosci 2010; 2: 12. 20552050 10.3389/fnagi.2010.00012 PMC2874397

[bib31] Maes M, Galecki P, Chang YS, Berk M. A review on the oxidative and nitrosative stress (O&NS) pathways in major depression and their possible contribution to the (neuro)degenerative processes in that illness. Prog Neuro-Psychopharmacol Biol Psych 2011; 35: 676–692. 10.1016/j.pnpbp.2010.05.004 20471444

[bib32] Frodl T, Bokde ALW, Scheuerecker J, Lisiecka D, Schoepf V, Hampel H et al. Functional connectivity bias of the orbitofrontal cortex in drug-free patients with major depression. Biol Psychiatry 2010; 67: 161–167. 19811772 10.1016/j.biopsych.2009.08.022

[bib33] MacQueen G, Frodl T. The hippocampus in major depression: evidence for the convergence of the bench and bedside in psychiatric research? Mol Psychiatry 2011; 16: 252–264. 20661246 10.1038/mp.2010.80

[bib34] Peper JS, Brouwer RM, Boomsma DI, Kahn RS. Poll HEH. Genetic influences on human brain structure: a review of brain imaging studies in twins. Hum Brain Mapp 2007; 28: 464–473. 17415783 10.1002/hbm.20398 PMC6871295

[bib35] Thomas RM, Hotsenpiller G, Peterson DA. Acute psychosocial stress reduces cell survival in adult hippocampal neurogenesis without altering proliferation. J Neuroscience 2007; 27: 2734–2743. 17360895 10.1523/JNEUROSCI.3849-06.2007 PMC6672591

[bib36] van Hille B, Richener H, Schmid P, Puettner I, Green JR, Bilbe G. Heterogeneity of vacuolar H(+)-ATPase: differential expression of two human subunit B isoforms. Biochem J 1994; 303(Pt 1): 191–198. 7945239 10.1042/bj3030191 PMC1137575

[bib37] Nelson N. Structure and pharmacology of the proton-atpases. Trends Pharmacol Sci 1991; 12: 71–75. 1827218 10.1016/0165-6147(91)90501-i

[bib38] Egashira Y, Takase M, Takamori S. Monitoring of vacuolar-type H+ ATPase-mediated proton influx into synaptic vesicles. J Neurosci 2015; 35: 3701–3710. 25716867 10.1523/JNEUROSCI.4160-14.2015 PMC6605559

[bib39] Ertunc M, Sara Y, Chung C, Atasoy D, Virmani T, Kavalali ET. Fast synaptic vesicle reuse slows the rate of synaptic depression in the CA1 region of hippocampus. J Neurosci 2007; 27: 341–354. 17215395 10.1523/JNEUROSCI.4051-06.2007 PMC6672081

[bib40] Lange C, Prenninger S, Knuckles P, Taylor V, Levin M, Calegari F. The H(+) vacuolar ATPase maintains neural stem cells in the developing mouse cortex. Stem Cells Dev 2011; 20: 843–850. 21126173 10.1089/scd.2010.0484 PMC3128780

[bib41] Talarowska M, Bobinska K, Zajaczkowska M, Su KP, Maes M, Galecki P. Impact of oxidative/nitrosative stress and inflammation on cognitive functions in patients with recurrent depressive disorders. Med Sci Monit 2014; 20: 110–115. 24457625 10.12659/MSM.889853 PMC3907532

[bib42] Wang YL, Floor E. Hydrogen peroxide inhibits the vacuolar H+-ATPase in brain synaptic vesicles at micromolar concentrations. J Neurochemistry 1998; 70: 646–652. 10.1046/j.1471-4159.1998.70020646.x 9453558

[bib43] Reiner AP, Hartiala J, Zeller T, Bis JC, Dupuis J, Fornage M et al. Genome-wide and gene-centric analyses of circulating myeloperoxidase levels in the charge and care consortia. Hum Mol Genet 2013; 22: 3381–3393. 23620142 10.1093/hmg/ddt189 PMC3723315

[bib44] Vaccarino V, Brennan ML, Miller AH, Bremner JD, Ritchie JC, Linclau F et al. Association of major depressive disorder with serum myeloperoxidase and other markers of inflammation: a twin study. Biol Psychiatry 2008; 64: 476–483. 18514165 10.1016/j.biopsych.2008.04.023 PMC2597204

[bib45] Pope SK, Kritchevsky SB, Ambrosone C, Yaffe K, Tylavsky F, Simonsick EM et al. Myeloperoxidase polymorphism and cognitive decline in older adults in the health, aging, and body composition study. Am J Epidemiol 2006; 163: 1084–1090. 16641309 10.1093/aje/kwj146

[bib46] Major Depressive Disorder Working Group of the Psychiatric GWAS Consortium Major Depressive Disorder Working Group of the Psychiatric GWAS Consortium Ripke S Major Depressive Disorder Working Group of the Psychiatric GWAS Consortium Wray NR Major Depressive Disorder Working Group of the Psychiatric GWAS Consortium Lewis CM Major Depressive Disorder Working Group of the Psychiatric GWAS Consortium Hamilton SP Major Depressive Disorder Working Group of the Psychiatric GWAS ConsortiumWeissman MM et al. A mega-analysis of genome-wide association studies for major depressive disorder. Mol Psychiatry 2013; 18: 497–511. 22472876 10.1038/mp.2012.21 PMC3837431

